# Supplementary material for: Expression of Microtubule-Associated Proteins in Relation to Prognosis and Efficacy of Immunotherapy in Non-Small Cell Lung Cancer
Source: Front Oncol. 2021 Oct 1;11:680402. doi: 10.3389/fonc.2021.680402 (PMC8517487; doi:10.3389/fonc.2021.680402)
Supplement: Supplementary file 1 [file DataSheet_1.zip › immunephenotype/IPS_TCGA-68-7755-01A-11R-2125-07.pdf]

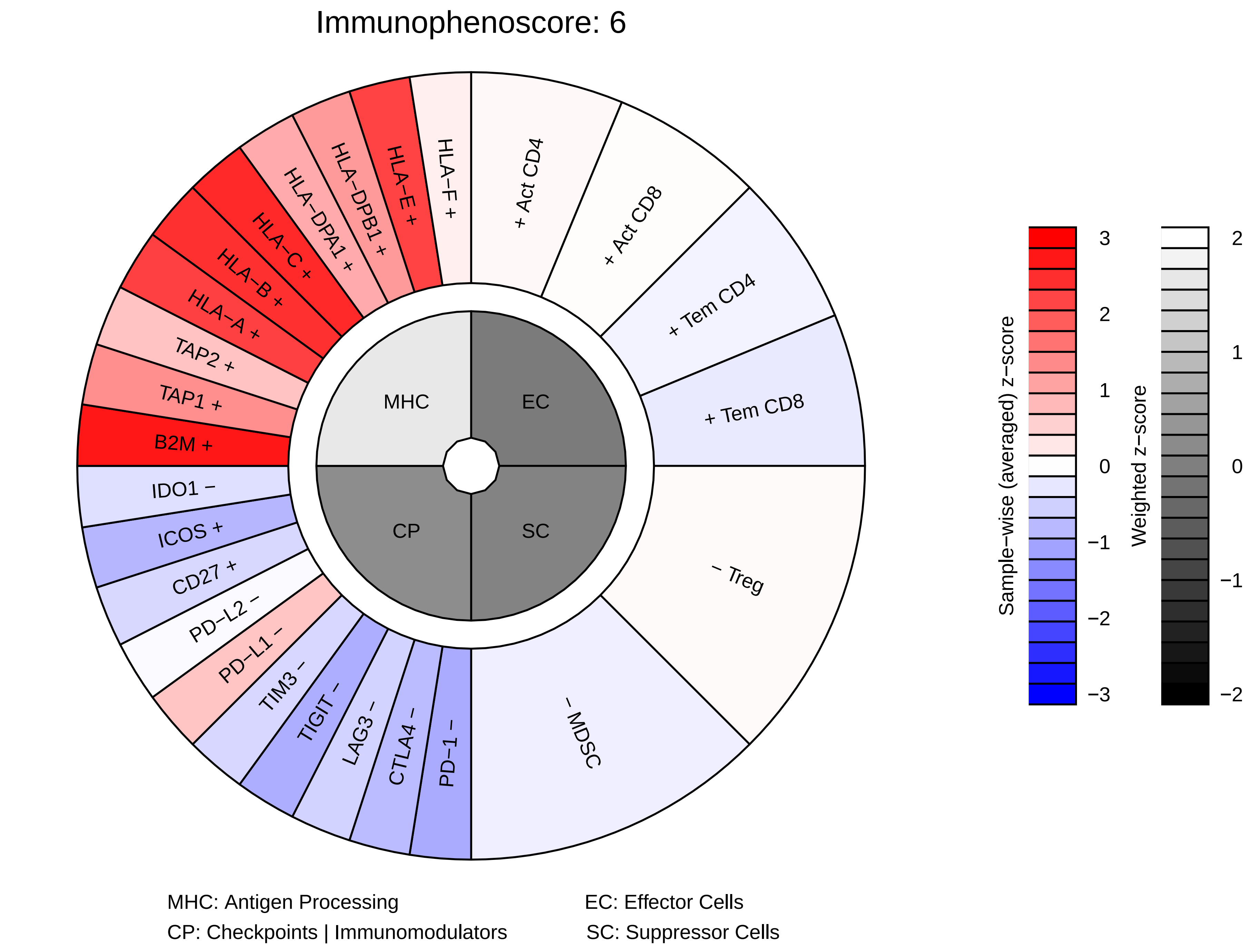
Immunophenoscore: 6
HLA–F +
HLA–E +
HLA–DPB1 +
HLA–DPA1 +
HLA–C +
HLA–B +
HLA–A +
TAP2 +
TAP1 +
B2M +
IDO1 –
ICOS +
CD27 +
PD–L2 –
PD–L1 –
TIM3 –
TIGIT –
LAG3 –
CTLA4 –
PD–1 –
– MDSC
– Treg
+ Tem CD8
+ Tem CD4
+ Act CD8
+ Act CD4
MHC
EC
CP
SC
Sample–wise (averaged) z–score
3
2
1
0
−1
−2
−3
Weighted z–score
2
1
0
−1
−2
MHC: Antigen Processing
EC: Effector Cells
CP: Checkpoints | Immunomodulators
SC: Suppressor Cells
